# Antimicrobial activities encountered by sulfur nanoparticles combating Staphylococcal species harboring scc*mec*A recovered from acne vulgaris

**DOI:** 10.3934/microbiol.2021029

**Published:** 2021-11-30

**Authors:** Noha M. Hashem, Alaa El-Din M.S. Hosny, Ali A. Abdelrahman, Samira Zakeer

**Affiliations:** 1 Ministry of Health, Cairo, Egypt; 2 Department of Microbiology and Immunology, Faculty of Pharmacy, Cairo University; 3 Department of Microbiology and Immunology, Faculty of Pharmacy, MTI University, Egypt; 4 Department of Microbiology and Immunology, Faculty of Pharmacy, Suez Canal University, Egypt

**Keywords:** Acne vulgaris, chitosan, sulfur nanoparticles, *Staphylococcus aureus*, *Staphylococcus epidermidis*

## Abstract

Over decades, sulfur has been employed for treatment of many dermatological diseases, several skin and soft tissue, and *Staphylococcus* infections. Because of its abuse, resistant bacterial strains have emerged. Nanotechnology has presented a new horizon to overcome abundant problems including drug resistance. Nano-sized sulfur has proven to retain bactericidal activity. Consequently, the specific aims of this study are exclusively directed to produce various sulfur nanoparticles formulations with control of particle size and morphology and investigate the antibacterial activity response specifically classified by the category of responses of different formulations, for the treatment of acne vulgaris resistant to conventional antibiotics. In this study, we produced uncoated sulfur nanoparticles (SNPs), sulfur nano-composite with chitosan (CS-SNPs), and sulfur nanoparticles coated with polyethylene glycol (PEG-SNPs) and evaluate their bactericidal impact against *Staphylococcus aureus* and *Staphylococcus epidermidis* isolated from 173 patients clinically diagnosed acne vulgaris. Accompanied with molecular investigations of *erm*B and *mec*A resistance genes distribution among the isolates. Sulfur nanoparticles were synthesized using acid precipitation method and were characterized by scanning electron microscope (SEM), transmission electron microscopy (TEM), energy dispersed x-ray spectroscopy (EDX), and Fourier transform infrared spectroscopy (FTIR). Moreover, agar diffusion and broth micro-dilution methods were applied to determine their antibacterial activity and their minimum inhibitory concentration. PCR analysis for virulence factors detection. Results: TEM analysis showed particle size of SNPs (11.7 nm), PEG-SNPs (27 nm) and CS-SNPs (33 nm). Significant antibacterial activity from nanoparticles formulations in 100% dimethyl sulfoxide (DMSO) with inhibition zone 30 mm and MIC at 5.5 µg/mL. Furthermore, the prevalence of *mecA* gene was the most abundant among the isolates while *ermB* gene was infrequent. Conclusions: sulfur nanoparticles preparations are an effective treatment for most *Staphylococcus* bacteria causing acne vulgaris harboring multi-drug resistance virulence factors.

## 1. Introduction

Acne Vulgaris is considered to be the most prevalent dermatological problem affecting 85% of young adults worldwide [1]. The most predominant organisms isolated from acne lesions include *Cutibacterium acne*, *Staphylococcus epidermidis* (*S.epidermidis*), *Malassezia furfur*, and *Staphylococcus aureus (S.aureus)* [1]. Significant evidence suggested a pathological role of *S.aureus* in acne pathogenesis. Widespread consumption of topical antibiotics has led to change of the sensitivity patterns to antibiotics and emerge of more virulent pathogens such as Methicillin-resistant *Staphylococcus aureus* (MRSA) [2]. In MRSA a mobile genetic elements (Staphylococcal cassette chromosome mec) is integrated into *S.aureus* chromosome, which is responsible for its resistance. In which the *mecA* gene encodes a distinct methicillin-resistant transpeptidase identified as penicillin-binding protein 2a (PBP2a). This protein exhibits low affinity to β-lactam antibiotics, which makes this phenotype resistant to all types of these drugs [3]. Along with the expression of macrolide-lincosamide-streptogramin B resistance (MLSB) typically to clindamycin and erythromycin. These drugs have been beneficial choices for treating skin and soft-tissue infections caused by staphylococci. The most predominant type of resistance is ribosomal binding site modification (by methylation or mutation in 23s rRNA gene) encoded by *erm* genes (erythromycin ribosome methylase) mediated by *ermA, ermB, and ermC* genes [4]. Therefore, restoring drug sensitivity is a substantial necessity that has a potential to be affordable by nanoscience.

Sulfur is biologically active element that has been used in dermatology for centuries. It has antibacterial, antifungal, antiviral, and Keratolytic activities besides its anti-tumor activity in the biomedicine field [5],[6]. Sulfur is used for management of different dermatological diseases such as, scabies, acne, and dandruff [7]. As a result, resistance to sulfur in elemental form has emerged. Recently, a few researches have been performed to synthesize sulfur nanoparticles and evaluate their antimicrobial activity [8]–[11].

Chitosan as well has received much scientific attention in the nanotechnology discipline in the field of drug delivery and tissue engineering. It has inimitable characteristics such as biocompatibility, biodegradability, bioavailability in addition to antibacterial activity [12]. It is a naturally occurring polysaccharide derived from crustaceans shells [13]. Chitosan exhibits more antibacterial activity against gram-positive bacteria over gram-negative bacteria [14].

Coating hydrophobic element as sulfur with chitosan or surfactant as PEG assists its dispersion and facilitates its cutaneous penetration. Therefore, this article is covering some aspects of antibacterial activity of sulfur nanoparticles specifically formulated for acne vulgaris treatment. The specific aims of this article are exclusively directed to produce various sulfur nanoparticles formulations with control of particle size and morphology and investigate their antibacterial impact on clinical isolates of *S.aureus* and *S.epidermidis* recovered from acne vulgaris lesions compared to elemental sulfur (ES). In addition to molecular analysis to investigate the prevalence rates of both *ermB* and *mecA* resistance genes in those isolates.

## 2. Methodology

### 2.1. Patients

This was a cross sectional study including 173 patients with clinically diagnosed acne who visited El Hod El Marsood Hospital and the Dermatology Clinic of El Khanka Hospital between March 2018 and December 2018. The study was approved by the Ethics Committee of Suez Canal University. First, patients were asked to fill out a questionnaire including questions about gender, age, duration of disease, history of acne treatment, and current or recent antibiotic use. Then swaps were collected from inflammatory pustules transferred into a thioglycolate transport medium (Merck, Darmstadt, Germany) and was ready for cultivation.

### 2.2. Bacterial isolation and identification

Each specimen was inoculated onto a plate of mannitol salt agar (MSA) (Himedia Laboratory M118-500G, Mumbai, India), and incubated at 37 °C aerobically up to 48 hrs. After incubation, MSA plates were examined for growth of *staphylococcus* species and a single colony was taken and cultured on nutrient agar (Himedia Laboratory M001-500G, Mumbai, India) and incubated at 37 °C aerobically for 24 hrs. Identification of gram-positive staphylococcal strains were carried out by conventional microbiological standard tests including mannitol fermentation activity, classical colony morphology, hemolysis on blood agar, and biochemical tests (catalase and coagulase).

### 2.3. Antibiotic susceptibility test

Using the disk diffusion method the susceptibility of *S. aureus* and *S. epidermidis* isolates were done and interpreted according to CLSI guidelines 2019 [15]. A bacterial suspension equivalent to 0.5 McFarland streaked over plates of Muller-Hinton agar (MHA) (Himedia Laboratory M173-500G, Mumbai, India), then antibiotic disks (Hi-Media Laboratories Pvt. Ltd. Mumbai, India) were applied. The antibiotic disks contain 30 µg tetracycline, 30 µg doxycycline, 15 µg erythromycin, 2 µg clindamycin, 10 units penicillin, 15 µg gentamycin, 10 µg fusidic acid, 5 µg ofloxacin, 30 µg cefoxitin and 15 µg chloramphenicol. The resulting plates were incubated aerobically at 37 °C for 18 hrs to 24 hrs and the inhibition zones were measured.

### 2.4. Synthesis and characterization of sulfur nanoparticles

#### 2.4.1. Materials

Sodium thiosulfate pentahydrate (STS) was purchased from Chemajet (Mandara, Alexandria, Egypt). Chitosan (derived from shrimp, C-02569/110 and degree of acetylation 93%) was purchased from Oxford Lab Chem (Mumbai, Maharashtra, India). Hydrochloric acid 30% was procured from El Salam Chemical Industry (SIC) (6-October, Egypt). Glacial acetic acid was obtained from Piochem (AC0121-Giza, Egypt). Poly ethylene glycol PEG-300 (Fluka, Sigma-Aldrich 90878, Germany) was kindly provided by Egyptian National Research Institute.

#### 2.4.2. Synthesis of sulfur nanoparticles

All nanoparticles formulations were prepared following the method of Shankar et al. [16]. For uncoated sulfur nanoparticles (SNPs) 0.567 g of STS Na_2_S_2_O_3._5H_2_O (MWT. 248.189/mole) was dissolved in 200 mL distilled water. Then 25mL 0.2 M HCl was added and the reaction mixture was kept for mechanically stirring at 1300 rpm for 40 minutes till equilibrium. After completing the reaction, the turbid suspension was added to electric mixer (7000 rpm,750 w) for 2 minutes, then it was transferred to water bath sonicator for additional 15 minutes. The nanoparticles were then centrifuged and the supernatant was discarded. SNPs were washed 7 to 8 times with milli_Q water until pH reached neutral. After that, SNPs were submitted for lyophilization.

For CS-SNPs 0.721 g of STS was dissolved in 253 mL distilled water, then 25 mL 0.2 M HCl was added and stirred at 1300 rpm for 20 minutes. Afterward, 15ml of chitosan solution (0.075 g chitosan MW. 161.16DA dissolved in 15 mL 1% v/v acetic acid) was added and stirred for 20 minutes. After that sonicated, centrifuged, and washed first with 1% v/v acetic acid then washed 6 to 7 times with milli-Q water till pH reached neutral. Then, submitted to lyophilization.

For the synthesis of PEG-SNPs, a 253 mL of 1% PEG-300 solution was added to 0.721 g of STS and stirred for 20 minutes followed by addition of HCl and proceeded until dry as mentioned before. Elemental sulfur was obtained from a reaction between 40 gm STS dissolved in 60 mL distilled water and 20mL 1M HCl, the resulted ppt was filtered and dried at 50 °C for 3 hrs [17].

#### 2.4.3. Characterization of sulfur nanoparticles formulations

Particle size and surface morphology of elemental sulfur (ES) was observed by SEM (Model: JSM-5500 LV; JEOL Ltd-Japan). All SNPs formulations were admitted to TEM. The image analysis was performed using JEOL-1010 TEM instrument (Tokyo, Japan) operated at an accelerating voltage of 200 kV. Elemental analysis, for the synthesized SNPs, was performed using EDX on SEM instrument (Model: JSM-5500 LV; JEOL Ltd-Japan) for evaluation of composition and purity. FTIR analysis was performed to confirm surface modifications. For CS-SNPs, a spectra was recorded using ATR-FTIR method. For PEG-SNPs, we employed potassium bromide beads method [9].

### 2.5. Evaluation of antibacterial activity of the nanoparticles against isolates of Acne Vulgaris

We used two different solvents to prepare the nanoparticle suspensions. Distilled water (with 1% poly-N-vinylpyrrolidone (PVP) (MW. ~40000) (Loba Chemie, Mumbai, India)) for uncoated SNPs (SNPs/H), PEG-SNPs (PEG-SNPs/H), and CS-SNPs (CS-SNPs/H). In addition to 100% DMSO for uncoated SNPs (SNPs/D) and PEG-SNPs (PEG-SNPs/D). The antibacterial effect of the nanoparticles was tested by both the agar diffusion method and broth microdilution method according to CLSI guidelines 2019 [15].

#### 2.5.1. Agar diffusion method

Bacterial suspension was adjusted to 0.5 McFarland and inoculated into a MHA plates. The concentration of the nanoparticles in DMSO was 3 mg/mL and in water was 8 mg/mL. Aliquots of 50 µL of each nanoparticle were added to 6 mm wells made on MHA. All plates were incubated at 37 °C for 24 hrs and inhibition zones were measured [18].

#### 2.5.2. Broth microdilution method

The broth microdilution method is a precise antimicrobial test presenting a quantitative data on the antimicrobial activity of antimicrobial agents [16]. For each nanoparticles suspension aliquots of 50 µL of two-fold serially diluted nanoparticles were prepared in a 96-well microtiter plate to obtain final concentrations for nanoparticles formulations in water ranging from 31.25 to 4000 µg/mL, and final concentrations ranging from 5.469 to 700 µg/mL for nanoparticles in DMSO. Then 50 µL of nutrient broth was added followed by 50 µL of bacteria (5 x 10^5^ CFU/ML). Plates were then incubated at 37 °C for 15 hrs. To assess the viable bacteria remaining after incubation 20 µL of resazurin (0.4 mg/mL solution) (Sigma-Aldrich, St. Louis, MO, USA) was added to each well and incubated for 3 hrs. After incubation the MIC was determined using resazurin colorimetric assay, in which resazurin (blue) was reduced by viable cell into resorufin (pink). Each microtiter plate was used for evaluating five nanoparticles suspensions against two bacterial isolates (Figure 8).

### 2.6. Genotypic detection of ermB and mecA genes

DNA was extracted from 101 isolates (31 *S.aureus and 70 S.epidermidis*) using Gene JET Genomic DNA purification kit according to the manufacturer's instructions (Thermo Scientific). After DNA extraction, PCR was performed in 25 µL reaction volumes containing 12.5 µL DreamTaq master mix PCR Kit (Thermo scientific, USA), 0.5 µL of each primer, and 3 µL of DNA template using a thermocycling profile. The primers used in this study are presented in Table 1, in which we have designed the primers for *ermB* using Primer-BLAST tools. After amplification, PCR products were detected by electrophoresis on a 1.5% agarose gel. The gel was then visualized in UV light and digital images made of the gel by Gel Documentation Systems (Bio-Rad) [19].

**Table 1.** Primers and conditions used in the PCR.

Gene	Primer name	Direction	Size (bp)	Primer sequence (50–30)	Ref	PCR conditions
*mec*A	*mec*A-F	FWD	500	5′-AAAATCGATGGTAAAGGTTGGC-3′	[20]	Denaturation step at 95 °C for 4 min, 35 cycles of 95 °C for 30 s, 55 °C for 45 s and 72 °C for 1 min, with a final extension step of 72 °C for 10 min.
	*mec*A-R	RVD	500	5′-AGTTCTGCAGTACCGGATTTG-3′		
*erm*B	*erm*B-F	FWD	299	5-AAGGGCATTTAACGACGAAACTG-3		
	*erm*B-R	RVD	299	5-TCGGTGAATATCCAAGGTACGC-3		

## 3. Result

### 3.1. Isolation and identification of bacterial isolates

Out of 173 specimens, 31 (18%) were *S.aureus*, 70 (40%) were *S.epidermidis*, and 72 (42%) were other organisms. All *S.aureus* showed positive catalase, coagulase, and mannitol fermentation activities while *S.epidermidis* showed positive catalase and negative coagulase and mannitol fermentation activities. Additionally, all *S.aureus* isolates in this study had no hemolytic activities.

### 3.2. Antibiotic susceptibility pattern of S.aureus and S.epidermidis isolates

**Figure 1.**
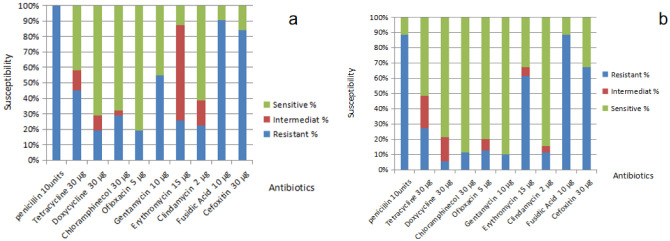
Antibiotic susceptibility pattern of (a) *Staphylococcus aureus* and (b) *Staphylococcus epidermidis* isolates recovered from acne vulgaris.

The distributions of the antibiotic susceptibilities are shown in (Figure 1). In which *S.aureus* isolates were more susceptible to ofloxacin with 80.6% followed by doxycycline, chloramphenicol, and clindamycin with 71%, 67.6% and 61.3%, respectively. It showed maximum resistance toward penicillin with 100% followed by fusidic acid, cefoxitin, gentamicin, tetracycline, and erythromycin with 90.3%, 83.9%, 54.8%, 45.2% and 25.8%, respectively. Whereas *S.epidermidis* isolates were more susceptible to gentamicin with 90% followed by chloramphenicol, clindamycin, ofloxacin, doxycycline, and tetracycline with 88.6%, 84.3%, 80%, 78.6% and 51.4%, respectively. It also showed maximum resistance toward penicillin and fusidic acid with 88.6% followed by cefoxitin and erythromycin with 75.7% and 61.4%, correspondingly.

### 3.3. Synthesis and characterization of sulfur nanoparticles

**Figure 2.**
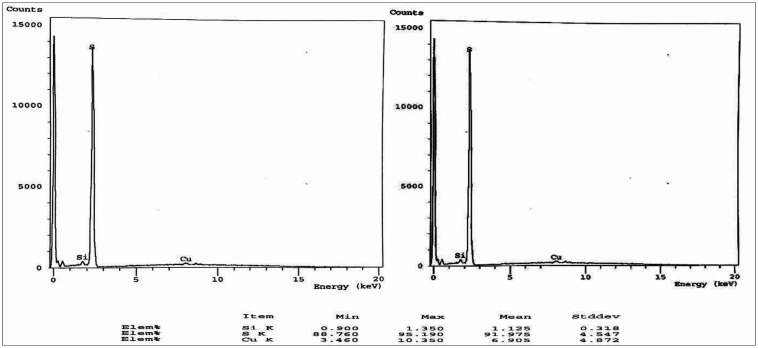
Energy dispersed x-ray spectroscopy of SNPs at two different positions indicating high purity with very small traces of copper and silicon.

**Figure 3.**
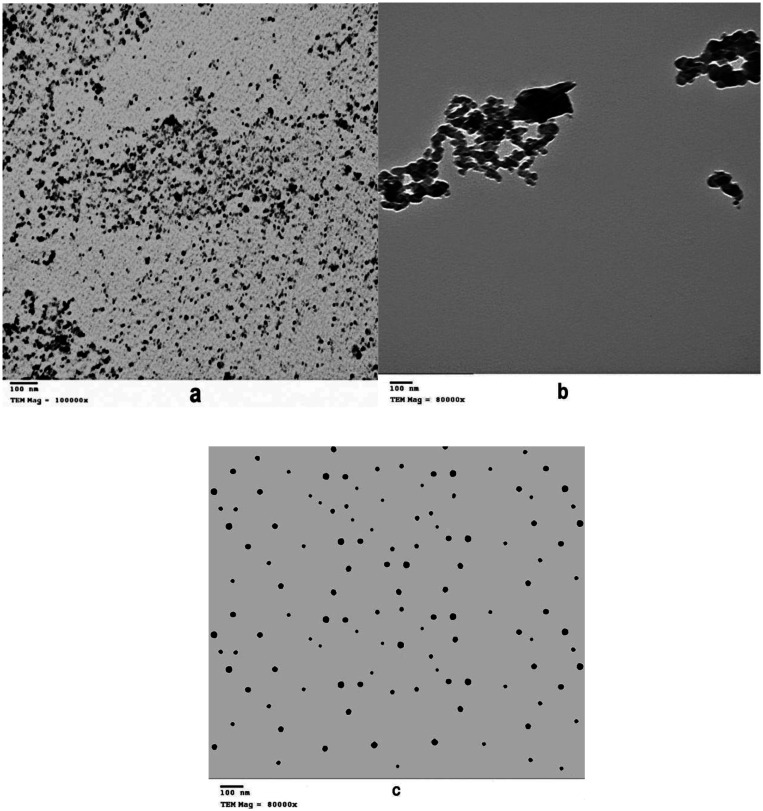
Transmission electron microscope image with a 100 nm scale bar. (a) Uncoated sulfur nanoparticles, (b) Sulfur nanoparticles coated with chitosan, (c) Sulfur nanoparticles coated with PEG.

**Figure 4.**
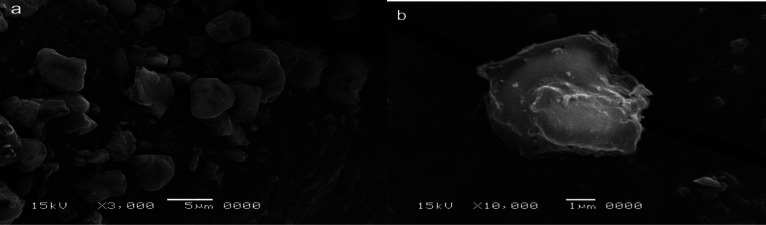
Characterization of elemental sulfur. (a) Scanning electron microscope image with a 5 µm scale bar. (b) Scanning electron microscope image with a 1 µm scale bar. Both images show uneven surface topography of the synthesized elemental sulfur with particle size more than 1 µm.

**Figure 5.**
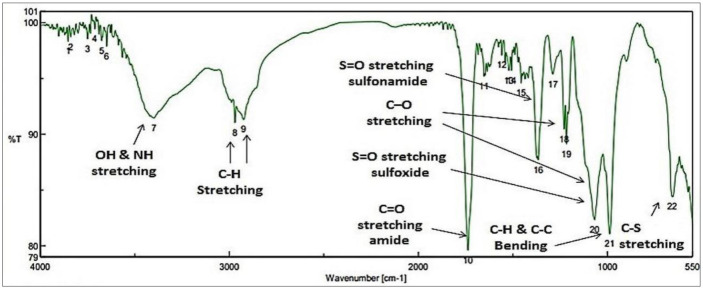
Fourier transform infrared spectrum of transmittance v/s wavenumber, depicting major functional groups of both chitosan and sulfur.

**Figure 6.**
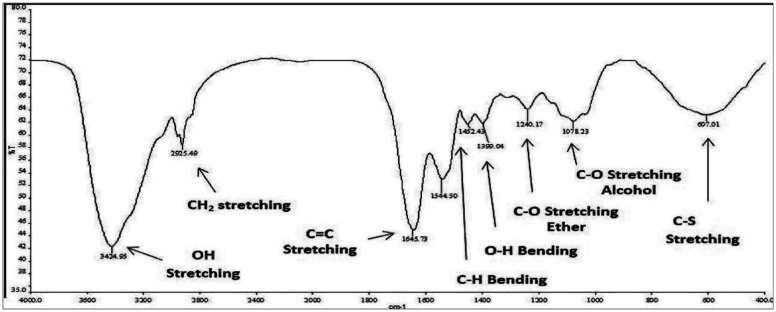
Fourier transform infrared spectrum of transmittance v/s wavenumber, depicting major functional groups and corresponding band attributes of both PEG-300 and sulfur.

In the synthesis process of SNPs color changes from a colorless solution of STS to turbid yellowish white color upon addition of HCL that is an indication of SNPs formation. EDX analysis revealed high purity of the synthesized SNPs at two different positions (Figure 2). The morphology and particle size of all sulfur nanoparticles were analyzed using TEM instrument. Uncoated SNPs showed very fine particle size and wide particle size distribution (~3–31.24 nm) (Figure 3a). While chitosan-SNPs showed spherical particles with wider particle size distribution (~17.6–68.58 nm) (Figure 3b). However, PEG-SNPs showed formation of uniform spherical particles with smaller particle size distribution (~15.48–32.89 nm) (Figure 3c). SEM micrograph of elemental sulfur showed sulfur particles present in uneven surface topography with particle size ranging from 3 µm to 22 µm (Figure 4a and 4b). Particle size distributions of all nanoparticles are shown in Table 2. Figure 5 shows FTIR spectra of chitosan-SNPs in which the absorption bands in the region 3450–3200 cm^−1^ with a peak at 3397 cm^−1^ were assigned to -OH and -NH stretching vibrations [16]. Bands attribute at 2969 and 2923 cm^−1^ were assigned to –CH symmetric and asymmetric stretching. These bands are characteristics of polysaccharide. The Absorption band at 988 cm^−1^ was ascribed to the saccharide structure of chitosan [16]. A new band appeared at 657 cm^−1^ this band can be assigned to the stretching vibration of the C–S bond and this constitutes a successful synthesis of chitosan-SNPs formulation [21]. Figure 6 shows FTIR spectra of PEG-SNPs in which the absorption bands in the region 3450–3200 cm^−1^ with a peak at 3424.95 cm^−1^ were assigned to -OH stretching vibrations. The Absorption band at 1645 cm^−1^ was attributed to C=C stretching of PEG-300 [22]. Two bands appeared at 1452 and 1399 cm^−1^ were assigned to C-H bending and O-H bending, respectively. A new band centered at 657 cm^−1^, which was observed in the chitosan-SNPs spectrum. This could be assigned as well to the stretching vibration of the C–S bond in the PEG-SNPs [21]. This provides a significant evidence for successful synthesis of PEG-SNPs formulation.

**Table 2.** Sulfur Nanoparticles size distribution using TEM analysis.

Nanoparticles	Size rang in nm	Mean diameter (nm)
Uncoated sulfur	3–31.24	11.74 ± 7.2*
Sulfur coated with PEG	15.79–32.89	27 ± 6.8*
Sulfur coated with chitosan	17.6–68.58	33.86 ± 10.2*

*± Standard deviation of mean diameter.

### 3.4. Evaluation of antimicrobial activity of sulfur nanoparticle

**Table 3.** Antibacterial activity of different formulations of sulfur nanoparticles presented as inhibition zones and MIC/MBC.

SNPs formulations	Inhibition zones in (mm)		MIC/MBC (µg/ml)	
	*S.aureus* N = 31	*S.epidermidis* N = 70	*S.aureus* N = 31	*S.epidermidis* N = 70
SNPs/D	29.76 ± 1.5	31 ± 3.6	5.469/43.7	5.469/43.7
PEG-SNPs/D	27 ± 2.65	26.3 ± 1.5	5.469/43.7	5.469/21.8
SNPs/H	28 ± 1	32 ± 3	31.25/125	31.25/125
PEG-SNPs/H	20 ± 1	20.3 ± 1.5	31.25/125	31.25/125
CS-SNPs/H	13.3 ± 1.5	15.33 ± 1.5	31.25/250	31.25/250

**Figure 7.**
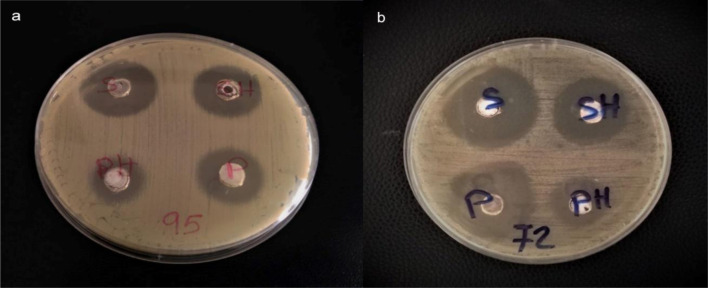
Agar diffusion method showed the antibacterial activity of different preparations of sulfur nanoparticles in form of inhibition zones. (a) *Staphylococcus aureus*. (b) *Staphylococcus epidermidis*. Well (S) represents SNPs/D with concentration 3 mg/mL, well (SH) represents SNPs/H with concentration 8 mg/mL, well (P) represents PEG-SNPs/D with concentration 3 mg/mL, and well (PH) represents PEG-SNPs/H with concentration 8 mg/mL.

**Figure 8.**
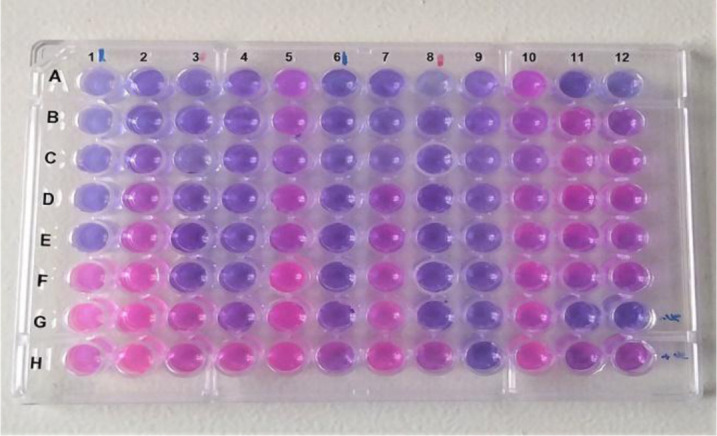
MIC of different nanoparticles formulations against *S.aureus* and *S.epidermidis* isolates. Columns 1–5 contained one isolate of *S.aureus*. Columns (6–10) contained one isolate of *S.epidermidis*. Columns 1 and 6 tested SNPs/D with concentrations (700–5.469 µg/mL), and columns 2 and 7 tested PEG-SNPs/D with the same concentrations. Columns (3 and 8) tested SNPs/H, columns (4 and 9) tested PEG-SNPs/H, and columns 5 and 10 tested CS-SNPs/H all with concentrations of 4000–31.25 µg/mL. columns 11 and 12 for both media and DMSO control in which columns 11 and 12 rows A–F. DMSO control with both bacteria starting from 50% dilution. Row G 11 and 12 negative media control and row H 11 and 12 positive media control.

In the current study, sulfur nanoparticles formulations showed characteristic antimicrobial activity against *S.aureus* and *S.epidermidis* as shown in Figure 7a, b. The nanoparticles formulations exhibited potent antimicrobial activity at lower concentrations while elemental sulfur in DMSO failed to exert antimicrobial activity at such concentrations and it managed to have a 12 mm inhibition zone at 20 mg/mL. We detect antimicrobial activity of SNPs formulations against *S.aureus* with inhibition zone ~30 mm and MIC at 5.469 µg/mL for sulfur formulations in 100% DMSO and inhibition zone ~28 mm and MIC at 31.25 µg/mL for sulfur formulations in water. And with inhibition zone ~31 mm and MIC at 5.469 µg/mL for sulfur formulations in 100% DMSO and inhibition zone ~32 mm and MIC at 31.25 µg/mL for sulfur formulations in water against *S.epidermidis*. As for CS-SNPs, inhibition zone was ~15 mm and the MIC reached to 31.25 µg/mL for both bacteria. Control to 100% DMSO in MHA plates showed no inhibition zones while it showed inhibitory effect in broth microdilution plates at 25% DMSO. Formulations of SNPs in 100% DMSO showed most potent and lower MIC compared to the same formulations in water (Table 3, Figure 8).

The results of inhibition zones are presented in mean value for clinical isolates with ±SD. Minimum inhibitory concentrations (MIC) and Minimum bactericidal concentrations (MBC) in µg/mL.

### 3.5. Cell viability assay

**Figure 9.**
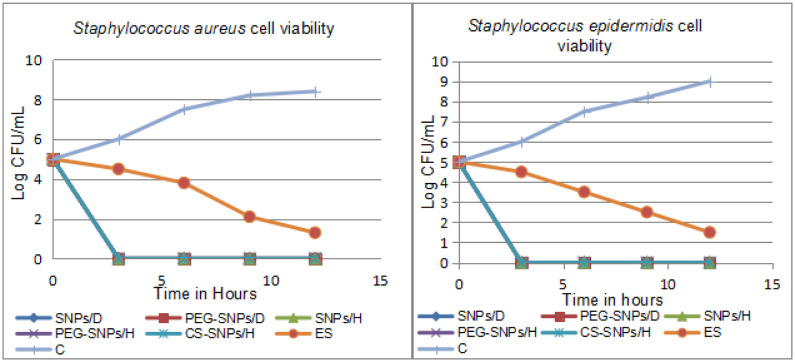
Cell viability assay versus time. Showing bactericidal activity of all sulfur nanoparticles formulations at high concentration after only 3 hrs incubation periods compared to the activity of elemental sulfur at a concentration of 20 mg/mL which showed a slight reduction in the viable count. The concentration of SNPs/D and PEG-SNPs/D were 0.7 mg/mL while the concentrations of SNPs/H, PEG-SNPs/H, and CS-SNP/H were 4 mg/mL. C is positive bacterial growth.

The change in the viable cells by the SNPs formulations over time was the same in both *S.aureus* and *S.epidermidis* shown in Figure 9. The antimicrobial activities of SNPs were evaluated using a total colony count. All SNPs formulations showed reduction in the viable count after only 3hrs incubation where elemental sulfur showed a slow reduction in the viable count but not completely reached zero at time interval 12 hrs.

### 3.6. Genotypic detection of ermB and mecA genes

#### 3.6.1. Detection of *mecA* gene

**Figure 10.**
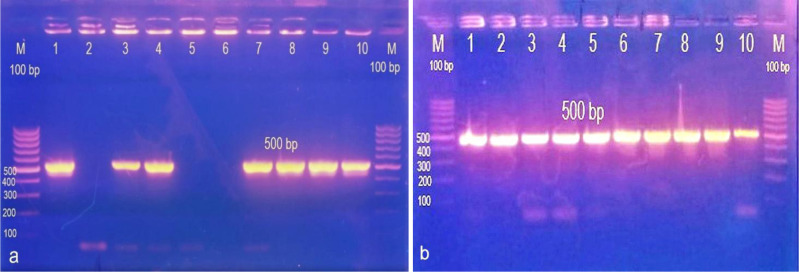
Amplicon of *mec*A gene; lane M:100 bp molecular weight ladder; lane 1 to 10 are tested isolates with positively amplified *mecA* as indicated by 500 bp PCR amplicon. (a) *S.epidermidis*, (b) *S.aureus*.

PCR was performed on 101 isolates (31 *S.aureus* and 70 *S.epidermidis*) for detection of *mec*A gene and *erm*B gene. Figure 10 showed the Agarose gel electrophoresis of PCR product amplified from *mecA* gene for *S.aureus* and *S.epidermidis*. In the current study, phenotypic methods applied on *S.aureus* revealed that 26 isolates (83.8%) were identified as MRSA and 5 isolates (19.2%) were identified as MSSA (methicillin- sensitive *S. aureus)* in which *mec*A gene was recovered from all MRSA isolates and was negative in MSSA isolates. Likewise *S.epidermidis* phenotypic methods using cefoxitin disc diffusion revealed that 53 isolates (75.7%) were MRS (methicillin-resistant staphylococci) and 17 isolates (24.3%) were MSS (methicillin-sensitive staphylococci), in which *mec*A was recovered from 48/53 (90.5%) MRS while the remaining 5/53 (9.4%) failed to produce the band of 500 bp specific for *mecA* gene (Table 4).

**Table 4.** Distribution of *mecA* gene and *ermB* gene among clinical isolates.

Resistant genes	*S.aureus*		*S.epidermidis*	
	MRSA* (n = 26)	MSSA* (n = 5)	MRS* (n = 53)	MSS* (n = 17)
*mec*A	26 + ve	5 - ve	48 + ve; 5-ve	17 -ve
*erm*B	1 + ve	-----	4 - ve	-----

*MRSA: methicillin resistant *S.aureus*, *MSSA: methicillin sensitive *S. aureus*, *MRS: methicillin resistant staphylococci, *MSS: methicillin sensitive staphylococci.

#### 3.6.2. Detection of *ermB* gene

**Figure 11.**
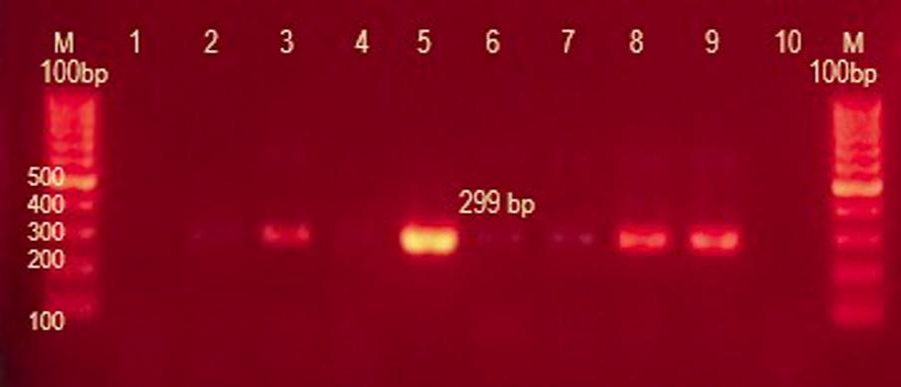
Amplicon of *ermB* gene; lane M:100 bp molecular weight ladder; lane 3,5,8, and 9 are tested isolates with positively amplified *erm*B as indicated by 299 bp PCR amplicon.

The *erm*B gene in this study was recovered from 1 (3.2%) MRSA isolate which was multidrug resistant and was negative in the rest of *S.aureus* isolates. It was also recovered in only 4 isolates (5.7%) of *S.epidermidis* and 66/70 failed to produce the band of 299 bp specific for *erm*B gene (Figure 11, Table 4).

## 4. Discussion

In the current study, all *S.aureus* isolates show no hemolytic activities on sheep blood agar. This phenomena was discussed recently by Zhang et al. 2016 in which, a strain of *S.aureus* with an incomplete hemolytic phenotype (SIHP) was isolated from clinical samples that SIHP strains retain potential extreme virulence [23],[24]. The resistance patterns of *S.epidermidis* against most common prescribed antibiotics for acne was variable in which it showed increased resistance to fusidic acid 88.6% and erythromycin 61.4% followed by tetracycline and clindamycin with rates 27.1% and 11.4%, respectively. Moon et al. 2012 determined that *S.epidermidis* strains were frequently resistant to erythromycin, tetracycline, and doxycycline with rates of 58.3, 30.6, and 27.3%, respectively [25]. In another study performed by Nakase et al. 2014 found that *S. epidermidis* was highly resistant to erythromycin and clarithromycin (58.6%) [26]. Nonetheless, in our study, *S.aureus* isolates exhibited high resistance to penicillin, fusidic acid, cefoxitin, gentamycin, Tetracycline, erythromycin, and clindamycin. Moon et al. 2012 observed that *S.aureus* from acne lesions showed the highest resistance to tetracycline, doxycycline, and erythromycin with values of 87.5, 87.5, and 75%, respectively [25]. An additional study by Doss et al. 2016 revealed that *S.aureus* isolates from acne vulgaris showed high resistance to minocycline, erythromycin, and doxycycline [27]. Consequently, our study aimed to synthesize various formulations of sulfur nanoparticles SNPs with controlled size and morphology for combating such a resistant bacteria causing acne vulgaris as alternative to conventional antibiotics. There are several parameters that control particle size and morphology, and these include, the reagent's concentration, temperature of the reactions, the velocity at which the reaction occurred, and sonication. Those help to control precipitations and crystal growth formation and also produce particles with a smaller size range. In our study, we produced smaller size range also by using smaller concentrations of HCL. The size range of the uncoated SNPs formed was (3–31.24 nm) with a mean diameter of (11.74 ± 7.2 nm) while in a previous study performed by Shankar et al. 2018 the size of non-caped sulfur nanoparticles were not uniform ranging from 10–70 nm with average diameter 45 ± 15.55 nm [16]. A similar study by Massalimov et al. 2012 showed sulfur nanoparticles with an average size of 20–25 nm obtained by using the acid decomposition of potassium polysulfide [28]. In our study, CS-SNPs were obtained with spherical shape and particle size ranging from (17.6–68.58 nm) with a mean diameter of (33.86 ± 10.2 nm). However Shankar et al. 2018 managed to use chitosan as a capping agent, the SNPs were uniform in size, and the size was in the range of 10–30 nm with a mean diameter of 15.80 ± 7.82 nm [16]. In a similar study by Y.H. Kim, et al. 2020 the shape of the SNPs was spherical, and the size was in the range of 10–80 nm [17]. PEG-SNPs in our study exhibited uniform spherical shape and smaller size distribution (~15.48–32.89 nm) with a mean diameter of 27 ± 6.8 nm. In a study performed by Choudhury et al. 2011 surface-modified SNPs of two different sizes were prepared via a modified liquid phase precipitation method, using sodium polysulfide and ammonium polysulfide as starting material and polyethylene glycol-400 (PEG-400) as the surface stabilizing agent. The result of TEM analysis revealed that an average particle size of ∼20 and ∼50 nm for SNP-1 and SNP-2, respectively [22]. Although the antibacterial activity of sulfur-nanoparticles has been proven, not many researches are performed for their evaluation. Shankar et al. 2018 reported antibacterial activity of SNPs against gram-negative and gram-positive bacteria in which chitosan capped SNPs showed MIC at 16 µg/mL against *E.coli* and *S.aureus*. Uncapped SNPs showed MIC at 256 µg/mL and 128 µg/mL against *E.coli* and *S.aureus* respectively [16]. Deshpande et al. 2008 reported that SNPs at 150 µg/mL exhibited the inhibition zones of 30 mm for *S.aureus* and 25 mm for *P. aeruginosa* [29]. Recently, Kim et al. 2020 showed also antibacterial activity of SNPs with MIC at 10mM for *S.aureus* [17]. Another study by Suleiman et al. 2015 demonstrated antibacterial activity of sulfur nanoparticles in DMSO against *S.aureus* at concentration 5.4 µg/mL but he did not correlate this activity to sulfur nanoparticles as control to DMSO showed inhibitory effect at 6.25 to 50% DMSO [30]. In our study, the nanoparticles formulations exhibited significant antimicrobial activity against *S.aureus* and *S.epidermidis*. Whereas SNPs formulations in 100% DMSO showed the most inhibitory effect against the isolates while 100% DMSO alone failed to inhibit such bacteria. DMSO may facilitate the penetration of SNPs into the bacterial cell and help to exert its lethal effect. Detection of *mecA* gene is a huge evidence for confirmation of MRSA isolates which in this study confer cefoxitin resistance. This statement was approved by several researches worldwide in Thailand [31], India [32], Sudan [33], Australia [34]. Likewise *S.epidermidis* phenotypic methods using cefoxitin disc diffusion revealed that 53 isolates (75.7%) were MRS and 17 isolates (24.3%) were MSS in which *mec*A was recovered from 48/53 (90.5%) MRS while the remaining 5/53 (9.4%) failed to produce the band of 500 bp specific for *mecA* gene. This finding is in agreement with a previous study in Nigeria reported Nineteen (19) of the 24 *mecA*-CoNS (Coagulase-negative staphylococci) were oxacillin sensitive [20]. Another study in UK reported that one *mecA* negative CoNS isolate was considered resistant phenotypically [35]. Additionally a previous study in Nigeria reported the complete absence of five major staphylococcal cassette chromosome mec (SCCmec) types and *mecA* genes as well as the gene product of the altered penicillin-binding protein (PBP2a) in isolates which were phenotypically MRSA suggesting a probability of hyper-production of *β*- lactamase as a cause of the phenomenon [36]. These findings afforded clear evidence for presence of other mechanisms exhibited by the organisms rather than harboring of *mecA* gene responsible for beta-lactam resistance of MRSA and CoNS. Consequently, molecular methods alone are not sufficient for confirmed classification of MRSA and CoNS isolates. Nevertheless, the occurrence of *erm* genes is fluctuating in different studies. For instance a study conducted by El-Mahdy et al. 2010 has detected the prevalence of *erm* genes in CoNS isolates from acne patients in which the tested isolates harbor *erm*(C) gene and *msr*(A) while *erm*A and *erm*B have not been detected [37]. Another study conducted by Gatermann et al. 2007 in which most (63%) erythromycin-resistant isolates harbored constitutively expressed *erm*(C) as the sole resistance determinant while the *erm*(A) and *erm*(B) determinants were comparatively rare [38]. This finding is compatible with our result in which the occurrence of ermB resistance gene is infrequent compare to the other genes.

## 5. Conclusions

Sulfur nanoparticles formulations (SNPs) were synthesized by liquid phase precipitation of sodium thiosulfate using low concentration of hydrochloric acid with/without the presence of PEG-300 as surfactant and chitosan. The nanoparticles exhibited significant antimicrobial activity against *multi-drug resistant S.aureus* and *S.epidermidis* recovered from acne vulgaris. These isolates exhibited high expression of *mec*A gene with infrequent expression of *erm*B gene. By using DMSO as a solvent for sulfur nanoparticle formulations, a bactericidal effect was achieved at low concentration. Assuming that sulfur exert its antibacterial activity through disruption of bacterial cell wall and leakage of cell organelles [39], solubility of sulfur nanoparticles in DMSO provides excessive penetration capacity with lower concentration for bacterial mortality. And this provides an effective treatment option for controlling acne vulgaris.
